# Holistic profiling of the venom from the Brazilian wandering spider *Phoneutria nigriventer* by combining high-throughput ion channel screens with venomics

**DOI:** 10.3389/fmolb.2023.1069764

**Published:** 2023-02-14

**Authors:** F. C. Cardoso, A. A. Walker, G. F. King, M. V. Gomez

**Affiliations:** ^1^ Institute for Molecular Bioscience, The University of Queensland, Brisbane, Australia; ^2^ Centre of Excellence for Innovations in Peptide and Protein Science, The University of Queensland, Brisbane, Australia; ^3^ Department of Neurotransmitters, Institute of Education and Research, Santa Casa, Belo Horizonte, Brazil

**Keywords:** spider venom, *phoneutria*, ion channel, high throughput screen, bioassays, proteomics, venomics, peptide drug

## Abstract

**Introduction:** Spider venoms are a unique source of bioactive peptides, many of which display remarkable biological stability and neuroactivity. *Phoneutria nigriventer*, often referred to as the Brazilian wandering spider, banana spider or “armed” spider, is endemic to South America and amongst the most dangerous venomous spiders in the world. There are 4,000 envenomation accidents with *P. nigriventer* each year in Brazil, which can lead to symptoms including priapism, hypertension, blurred vision, sweating, and vomiting. In addition to its clinical relevance, *P. nigriventer* venom contains peptides that provide therapeutic effects in a range of disease models.

**Methods:** In this study, we explored the neuroactivity and molecular diversity of *P. nigriventer* venom using fractionation-guided high-throughput cellular assays coupled to proteomics and multi-pharmacology activity to broaden the knowledge about this venom and its therapeutic potential and provide a proof-of-concept for an investigative pipeline to study spider-venom derived neuroactive peptides. We coupled proteomics with ion channel assays using a neuroblastoma cell line to identify venom compounds that modulate the activity of voltage-gated sodium and calcium channels, as well as the nicotinic acetylcholine receptor.

**Results:** Our data revealed that *P. nigriventer* venom is highly complex compared to other neurotoxin-rich venoms and contains potent modulators of voltage-gated ion channels which were classified into four families of neuroactive peptides based on their activity and structures. In addition to the reported *P. nigriventer* neuroactive peptides, we identified at least 27 novel cysteine-rich venom peptides for which their activity and molecular target remains to be determined.

**Discussion:** Our findings provide a platform for studying the bioactivity of known and novel neuroactive components in the venom of *P. nigriventer* and other spiders and suggest that our discovery pipeline can be used to identify ion channel-targeting venom peptides with potential as pharmacological tools and to drug leads.

## Introduction

Venomous animals are a highly adapted group of organisms whose evolutionary success excelled with the emergence of venom. Spider venoms, in particular, are rich in peptide knottins specialized in modulating, often with high potency and selectivity, voltage-gated ion channels that regulate the physiology of neuronal, muscular and cardiac systems (Cardoso and Lewis, 2018; Cardoso, 2020). Although such effects can be deleterious to envenomated animals, venom components can be tailored to selectively modulate ion channels in pathways of complex diseases such as chronic pain, motor neuron disease, and epilepsy. This has been demonstrated for numerous spider venoms (Smith et al., 2015; Cardoso and Lewis, 2018, 2019), including the venom of the infamous South American ctenid spider *Phoneutria nigriventer*, often referred as Brazilian wandering spider, banana spider or “armed” spider (Peigneur et al., 2018). Besides its clinical relevance due to frequent envenomation cases in Brazil, with approximately 4,000 cases per year (Isbister and Fan, 2011; Gewehr et al., 2013), *P. nigriventer* venom contains peptides that have therapeutic effects in a range of disease models including chronic pain (Pedron et al., 2021; Cavalli et al., 2022), Huntington’s disease (Joviano-Santos et al., 2022), glaucoma (da Silva et al., 2020) and erectile dysfunction (Nunes da Silva et al., 2019).

Initial studies of *P. nigriventer* venom employed fractionation *via* gel filtration and reversed-phase chromatography to separate the venom into five distinct groups of peptides based on their molecular weight and hydrophobicity properties; these groups were named PhTx1 to PhTx5 (Peigneur et al., 2018). PhTx1–4 comprise cysteine-rich peptides that are active on voltage-gated calcium (Ca_V_), sodium (Na_V_) and potassium (K_V_) channels, while PhTx5 is comprised of short linear peptides, with a total of 34 peptides identified (Peigneur et al., 2018). Proteotranscriptomic studies of *P. nigriventer* venom revealed additional peptides with high similarity to those previously described, but very few have been characterised pharmacologically (Cardoso et al., 2003; Richardson et al., 2006). This represents an obstacle to the exploration of the therapeutic potential of *P. nigriventer* venom.

Advances in venom-peptide research have yielded high-throughput cellular screens for the discovery and pharmacological characterisation of naturally occurring molecules with activity at ion channels and receptors in physiological pathways (Cardoso et al., 2015; Cardoso et al., 2021). These methods require only a small amount of venom compared to more traditional methods and allow the identification of therapeutically relevant peptides in the early stages of the screening. Besides drug development applications, these same bioassays can assist in unravelling the bioactivity of crude and fractionated venoms from biomedically relevant venomous animals to support studies of evolution and antivenom development, but much work remains to be done in this field.

This study aimed to provide a proof-of-concept in applying high-throughput cellular screens for multiple neuronal ion channels along with proteomic studies of fractionated venom to rapidly characterise spider venoms in terms of bioactive components. It was anticipated that such a pipeline would support envenomation and evolutionary studies and the development of therapeutics from animal venoms. The venom of *P. nigriventer* was selected as a model system due to its medical relevance, the considerable number of therapeutically relevant peptides already uncovered in the venom, and the wide knowledge base available. Our approach enabled identification of potent modulators of voltage-gated ion channels which were classified into four families of neuroactive peptides based on their activity and structures. In addition to the previously characterised neuroactive peptides in the *P. nigriventer* venom, we identified 27 additional cysteine-rich venom peptides in which neuroactivities are underexplored. This work contributes to the on-going discovery and structure-function characterisation of spider-venom peptides. Moreover, our bioassay pipeline can be used to guide future research into the discovery of venom peptides that modulate the activity of ion channels, and their development as pharmacological tools and drug leads.

## Materials and methods

**FIGURE 1:**
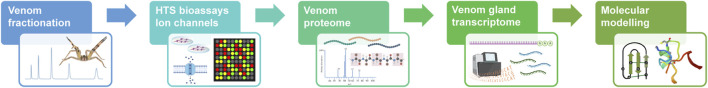
Flowchart of the venom peptide discovery pipeline applied in this study. Expanding from the traditional assay-guided fractionation, we applied HTS bioassays to characterize the pharmacology of venom peptides on multiple ion channels, followed by the identification of peptide masses and primary sequences using proteome and transcriptome. Ultimately, the three-dimensional structure of venom peptides was determined using *in silico* molecular modelling.

We applied a holistic approach combining methods in high throughput screens for ion channels, venom proteome, venom gland transcriptome and modelling of peptides as described in Figure 1.

### Cell culture

The human neuroblastoma cell line SH-SY5Y was maintained at 37**°**C in a humidified 5% CO_2_ incubator in Roswell Park Memorial Institute (RPMI) medium supplemented with 15% foetal bovine serum (FBS) and 2 mM L-glutamine. Replicating cells were sub-cultured every 3–4 days in a 1:5 ratio using 0.25% trypsin/EDTA.

### Venom fractionation

Crude venom milked from male and female specimens of *P. nigriventer* was kindly provided by Prof. Marcus Vinicius Gomez from the Institute of Teaching and Research of Santa Casa de Belo Horizonte, Belo Horizonte, Brazil. Venom (lyophilised, 1 mg) was dissolved in 100 μL Milli-Q water containing 0.05% trifluoroacetic acid (TFA) (Auspep, VIC, AU) and 5% acetonitrile (ACN) and centrifuged at 5,000 × g for 10 min to remove particulates. Venom was fractionated by reversed-phase high performance liquid chromatography (RP-HPLC) using a C18 column (Vydac 4.6 mm × 250 mm, 5 μm, Grace Discovery Sciences, United States) with a gradient of solvent B (90% ACN in 0.045% TFA) in solvent A (0.05% TFA). The gradient was 5% B for 5 min, followed by 20%–40% solvent B over 60 min at a flow rate 0.7 mL min^−1^. Peaks were collected every minute, with fraction 1 eluted between 1 and 2 min and so on for the other fractions. Venom fractions were lyophilised before storage at –20°C.

### Calcium influx assays

Venom fractions were screened for neuroactivity at human (h) Na_V_, Ca_V_1, Ca_V_2 and the α7 subtype of the human nicotinic acetylcholine receptor (nAChR-α7) as previously described (Cardoso et al., 2015). Briefly, SH-SY5Y cells were plated at 40,000 cells per well in 384-well flat clear-bottom black plates (Corning, NY, United States) and cultured at 37**°**C in a humidified 5% CO_2_ incubator for 48 h. Cells were loaded with 20 μL per well Calcium 4 dye (Molecular Devices) reconstituted in assay buffer containing (in mM) 140 NaCl, 11.5 glucose, 5.9 KCl, 1.4 MgCl_2_, 1.2 NaH_2_PO_4_, 5 NaHCO_3_, 1.8 CaCl_2_ and 10 HEPES pH 7.4 and incubated for 30 min at 37**°**C in a humidified 5% CO_2_ incubator. For the hCa_V_1 assay, the dye was supplemented with 1 μM ω-conotoxin-CVIF (CVIF) to inhibit Ca_V_2, and in the hCav2 assay the dye was supplemented with 10 μM nifedipine to inhibit Ca_V_1. For the nAChR-α7 assay, the dye was supplemented with PNU-120596 (Sigma-Aldrich), a positive allosteric modulator of nAChR-α7. Venom fractions were assayed in singleton for each ion channel tested. Fluorescence responses were recorded using excitation at 470–495 nm and emission at 515–575 nm for 10 s to set the baseline, then 300 s after addition of 10% venom fraction serial diluted at 1, 1:10, and 1:100, and for a further 300 s after addition of 50 μM veratridine for hNa_V_, 90 mM KCl and 5 mM CaCl_2_ for hCa_V,_ and 30 μM choline for nAChR-α7.

### Proteomics

Venom fractions eluting between 10 and 45 min on RP-HPLC were analysed by mass spectrometry to investigate the masses and primary structures of their peptide components. Native mass determinations were carried out with 20% of each fraction dried by vacuum centrifuge and resuspended in 20 μL 1% formic acid (FA), followed by analysis using by liquid chromatography/tandem mass spectrometry (LC-MS/MS). For identification of primary structures, 20% of each peptide fraction was reduced and alkylated by adding 40 μL of reagent composed of 4.875 mL ACN, 4.5 mL ultrapure water, 0.5 mL 1M ammonium carbonate pH 11.0, 100 μL 2-iodoethanol and 25 μL triethylphosphine, and incubating for 1 h at 37°C. Samples were speed dried in a vacuum centrifuge, and digested with 40 ng/μL trypsin in 50 mM ammonium bicarbonate pH 8.0 and 10% ACN overnight at room temperature. Trypsin was inactivated by adding 50 μL solution containing 50% acetonitrile and 5% formic acid (FA), dried in speed vacuum centrifuge, and resuspended in 1% formic acid.

LC-MS/MS samples were loaded onto a 150 mm × 0.1 mm Zorbax 300SB-C18 column (Agilent, Santa Clara, CA, United States) on a Shimadzu Nano LC system with the outflow coupled to a SCIEX 5600 Triple TOF (Framingham, MA, United States) mass spectrometer equipped with a Turbo V ion source. Peptides were eluted using a 30 min gradient of 1%–40% solvent B (90% ACN/0.1% FA) in solvent A (0.1% FA) at a flow rate of 0.2 mL/min. For MS1 scans, *m/z* was set between 350 and 2,200. Precursor ions with *m/z* 350–1,500, charge of +2 to +5, and signals with >100 counts/s (excluding isotopes within 2 Da) were selected for fragmentation, and MS2 scans were collected over a range of 80–1,500 m*/z*. Scans were obtained with an accumulation time of 250 ms and a cycle of 4 s.

A database of possible peptide sequences produced in *P. nigriventer* venom glands was compiled using a published venom-gland transcriptome (Diniz et al., 2018), from which open reading frames (ORFs) longer than 30 amino acids were identified and translated by TransDecoder. A list of 200 common MS contaminants was added to the translated ORFs, which was used as a sequence database to compare to mass spectral data using the Paragon algorithm in Protein Pilot 2.2 software (AB SCIEX). We report only peptides for which more than two tryptic fragments were detected with >95% confidence, or where one tryptic fragment was detected, and a secretion signal peptide was predicted by SignalP5.0.

### Molecular modelling

Venom peptides identified in this study were selected based on their cysteine-rich scaffold and bioactivity, and their three-dimensional (3D) structure were predicted using the AlphaFold 2 algorithm (Jumper et al., 2021). All 3D structures displayed were from unrelaxed models ranked 1 for each peptide prediction. 3D structures were visualised and analysed using PyMol (Pymol, 2023).

### Data analysis

Fluorescence traces from singletons were evaluated using the Maximum-Minimum or Area Under the Curve values generated after addition of ion channel activator. Data were normalised against the negative control (PSS buffer control) and positive control (ion channel activator) for each assay and corrected using the response over baseline from 1 to 5 s. No statistical analyses were required in this study.

## Results

### Screening of *P. nigriventer* venom fractions

**FIGURE 2:**
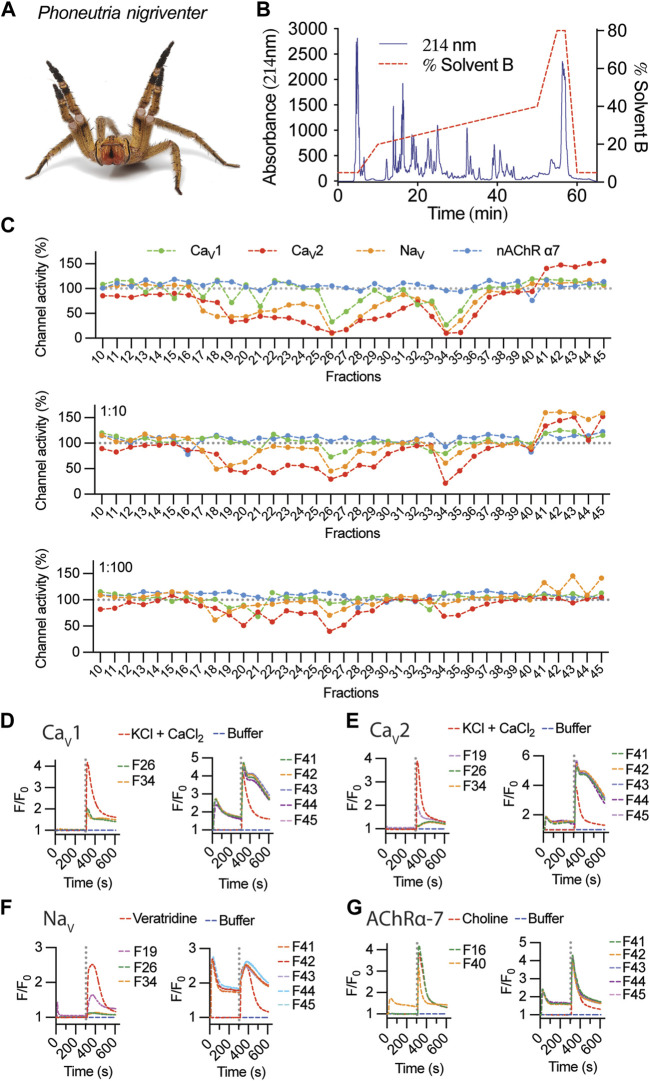
Fractionation and activity of *P. nigriventer* venom. **(A)** *P. nigriventer* specimen displaying threat posture (photo copyright Alan Henderson, www.minibeastwildlife.com.au). **(B)** RP-HPLC fractionation of 1 mg *P. nigriventer* venom. **(C)** Ion channel responses calculated from the area under the curve (AUC) after addition of selective activators for fractions 10 to 45, normalized to responses in the absence of venom fractions. **(D, E)** Representative fluorescence traces of the intracellular calcium responses of SH-SY5Y cells evoked by KCl + CaCl_2_ in the presence of venom fractions 26 and 34 for Ca_V_1, fractions 19, 26 and 34 for Ca_V_2, and fractions 41–45 for both Ca_V_1 and Ca_V_2 channels. **(F)** Representative fluorescence traces of the intracellular calcium responses of SH-SY5Y cells evoked by veratridine and in the presence of venom fractions 19, 26 and 34 and fractions 41–45. **(G)** Representative fluorescence traces of the intracellular calcium responses of SH-SY5Y cells evoked by choline and in the presence of venom fractions 16 and 40 and fractions 41–45. Grey dotted line indicates the KCl + CaCl_2_, veratridine or choline addition.

Fractionation of 1 mg of *P. nigriventer* (Figure 2A) crude venom using RP-HPLC produced numerous peaks eluting between 20% and 40% solvent B, and fractions eluting between 11 and 45 min were selected for pharmacological analysis (Figure 2B). Screening using the SH-SY5Y neuroblastoma cell line revealed strong modulation of voltage-gated ion channels including both inhibition or enhancement of ion channel activity (Figure 2C). Venom fractions eluting between 18 and 34 min showed strong inhibition of Ca_V_ and Na_V_ activity, while fractions eluting between 41 and 45 min strongly activated Ca_V_2 channels (Figure 2C, top panel). At a dilution of 1:10, these inhibitory effects persisted for both Na_V_ and Ca_V_2 channels for fractions eluting at 19–20 min and 26–34 min and was absent for Ca_V_1 channels (Figure 2C, middle panel). Fractions eluting from 21 to 25 min showed a clear preference for inhibiting only Ca_V_2 channels (Figure 2C). Interestingly, at 1:10 dilution, channel activity enhancement was stronger on Na_V_ channels compared to Ca_V_2 channels, suggesting potential concentration-dependent synergistic effects of venom peptides modulating both Na_V_ and Ca_V_2 channels. At the highest venom dilution of 1:100, persistent inhibition of Na_V_ channel was observed for fraction 20 (F20), while the remaining inhibitory fractions preferentially inhibited only Ca_V_2 channels (Figure 2C, bottom panel). Channel enhancement persisted for Na_V_ channels in fractions eluting from 41 to 45 min. No potent activity was observed against nAChR-α7 at any venom concentration tested. Overall, inhibitory activity was primarily observed for fractions eluting at shorter retention times (i.e., more hydrophilic compounds), while strong ion channel activation was induced by more hydrophobic peptides with longer RP-HPLC retention times.

Fluorescent traces measured upon addition of venom fractions revealed an increase in intracellular calcium ([Ca^2+^]_i_), suggesting that these venom peptides can activate closed channels as well as enhance the responses of these channels opened using pharmacological intervention (Figures 2D–G). This was observed for Ca_V_ responses in the presence of 1 μM CVIF (Ca_V_2 inhibitor, Figure 2D) and 10 μM nifedipine (Ca_V_1 inhibitor, Figure 2E). In the absence of Ca_V_ inhibitors, these [Ca^2+^]_i_ responses resemble the levels of Ca_V_1 responses in Figure 2D as observed for F40–F45 applied in the Na_V_ channels assay (Figure 2F). The activities of inhibitory fractions were mostly free from initial [Ca^2+^]_i_ responses upon venom addition, except for weak inhibitors observed in F19 for Na_V_ and F40 for nAChR-α7 (Figures 2F, G).

### Identification of peptides in *P. nigriventer* venom fractions

**FIGURE 3:**
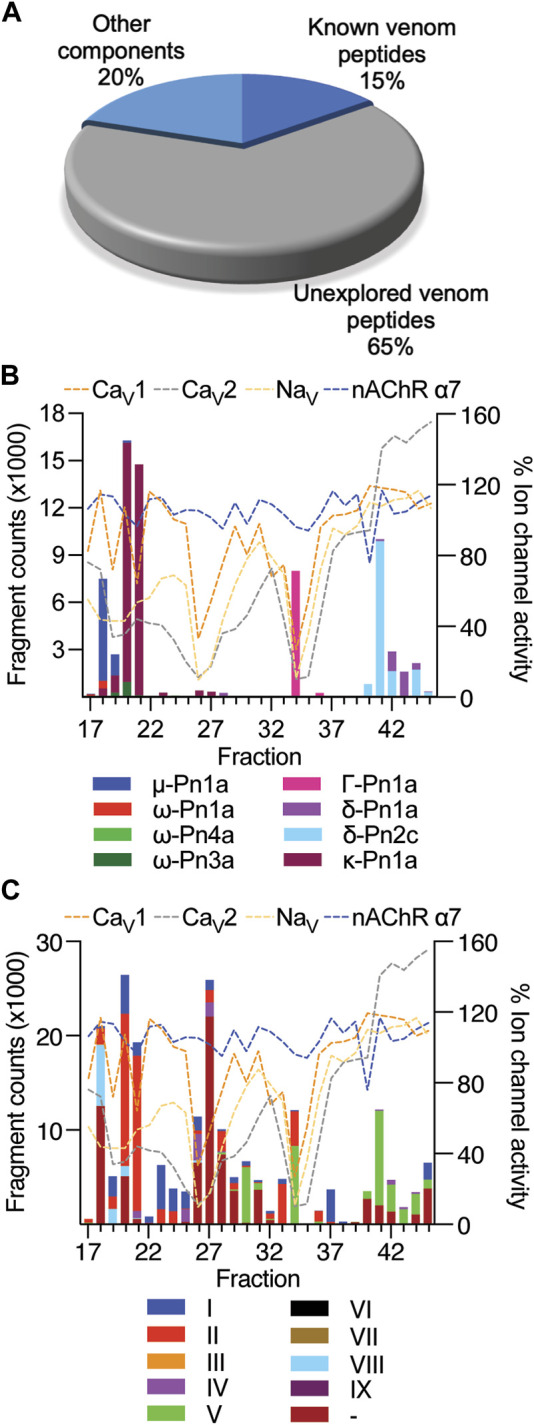
Estimated levels of peptide/protein venom components identified in fractions F17 to F45, and their respective bioactivity at Na_V_ and Ca_V_ channels and the nAChR-α7. **(A)** Proportion of known and unknown venom peptides and other venom components detected in this study. **(B)** Venom peptides with previously reported bioactivity detected in fractions by mass spectrometry and compared to fraction bioactivity at Na_V_ and Ca_V_ channels and the nAChR-α7. **(C)** Venom peptides detected in fractions classified according to their cysteine framework I to IX (Diniz et al., 2018), and compared to fraction bioactivity at Na_V_ and Ca_V_ channels and the nAChR-α7.

The venom of *P. nigriventer* has been extensively characterised in terms of composition and bioactivity (Diniz et al., 2018; Peigneur et al., 2018), including neuronal ion channel activity and proteomics, but not by using a combined approach. In this study, by combining these approaches, we were able to rapidly identify 58 peptides and proteins in the venom. Due to the complexity of previous nomenclature for *P. nigriventer* venom peptides, we refer to them here using both the rational nomenclature developed for spider toxins (King et al., 2008) and an identifying number (e.g., PN367) that is linked to a sequence and a list of previously used names in Supplementary Table S1. Of the 58 identified amino acid sequences, only eight (15%) are peptides that have had their bioactivity reported in previous studies (Figure 3A, Supplementary Table S1) (Peigneur et al., 2018). These included the known neuroactive components μ-CNTX-Pn1a (Tx1) (Diniz et al., 2006; Martin-Moutot et al., 2006), κ-CNTX-Pn1a (Tx3-1, PhK_V_) (Kushmerick et al., 1999; Almeida et al., 2011), ω-CNTX-Pn1a (Tx3-2) (Cordeiro Mdo et al., 1993), Γ-CNTX-Pn1a [Tx4(5-5)] (Paiva et al., 2016), δ-CNTX-Pn1a [Tx4(6-1)] (de Lima et al., 2002; Emerich et al., 2016), δ-CNTX-Pn2c (Tx2-5a) (Yonamine et al., 2004), ω-CNTX-Pn4a (Tx3-6) (Cardoso et al., 2003; Vieira et al., 2005) and ω-CNTX Pn3a (Tx3-4) (Dos Santos et al., 2002) (Figure 3B). Even among these eight peptides, only a few venom peptides have had their molecular pharmacology characterized in detail (Peigneur et al., 2018), or their activities confirmed using recombinant peptides (Diniz et al., 2006; Paiva et al., 2016; Garcia Mendes et al., 2021).

**FIGURE 4:**
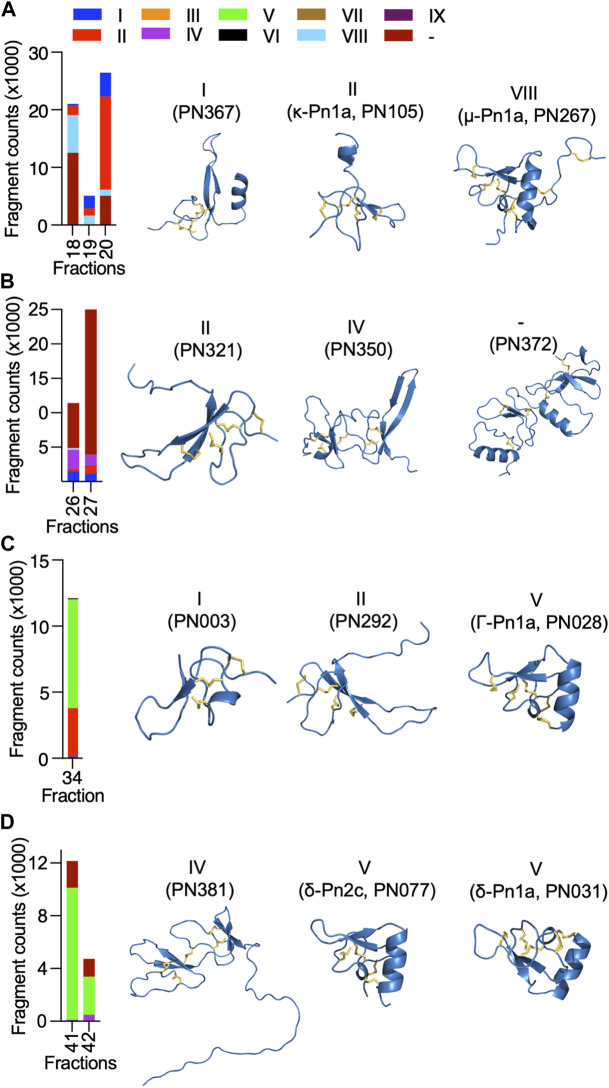
Diversity and estimated levels of cysteine-rich scaffolds identified in highly neuroactive RP-HPLC fractions from the venom of *P. nigriventer*, and their predicted 3D structures. **(A)** Fractions 18–20 comprised high levels of scaffolds I, II and VIII represented by the 3D structures of PN367, PN105 and PN267, respectively. **(B)** Fractions 26 and 27 comprised high levels of scaffolds II, and IV, and an undefined scaffold represented by the 3D structures of PN321, PN350 and PN372, respectively. **(C)** Fraction 34 comprised high levels of scaffolds I, II, and V represented by the 3D structures of PN003, PN292 and PN028, respectively. **(D)** Fractions 41 and 42 comprised high levels of scaffolds IV and V represented by the 3D structures of PN381, and PN077 and PN031, respectively.

Most of the identified sequences in this study (74%) represent peptides with unexplored bioactivity; 38 (65%) of the 43 peptides identified have cysteine-rich scaffolds typical of spider-venom peptides (Figure 3C). Some of these venom peptides, such as PN367 and PN363, have a type I scaffold (Diniz et al., 2018) and are predicted by Alphafold 2 to fold into cystine-knot scaffolds typical of spider-venom peptides (King and Hardy, 2013) (Figure 4). Scaffolds II-VIII either form elaborated cystine-knot folds with extra disulphide bonds, or alternative structures such as for scaffolds III and IV (Figure 4). Novel peptides with high identity with other toxins and not previously described in *P. nigriventer* venom included: PN367 displaying identity with a *Agelena orientalis* venom peptide; PN369 displaying identity with a *Lycosa singoriensis* venom peptide, and PN365 displaying scaffold III and identity with another *Lycosa singoriensis* venom peptide (Supplementary Table S1). Additional disulphide-rich scaffolds present in *P. nigriventer* venom include three peptides predicted by the algorithm HMMER to form a thyroglobulin type 1 repeat domain (E < e^−17^ in each case), one of which has been previously reported as U24-CNTX-Pn1a; peptide PN370 which displays high identity with a peptide found in venom of the scorpion *Scorpiops jendeki* and is predicted by the algorithm HMMER to form into a trypsin-inhibitor-like cysteine-rich domain (E < 2e^−13^); and the peptide PN376 that is predicted by HMMER to form a fungal protease inhibitor domain (E < e^−6^) (Supplementary Table S1). Additional new scaffolds identified in this study were named following the previous suggested nomenclature (Diniz et al., 2018) as X (CXCC motif, 12 Cys residues: −C−C−C−C−CXCC−C−C−C−C−C−), XI (12 Cys residues: −C−C−C−CXC−CXC−C−CXC−C−C−), XII (11 Cys residues: −C−C−CXC−CXC−C−C−CXC−C) and XIII (10 Cys residues: −C−C−C−C−C−C−CXC−C−C−), and include the peptides PN376, PN372, PN373 and PN375, and PN370, respectively.

Only 9% of the identified sequences were peptides with two or fewer Cys residues (Supplementary Table S1). F17 contained a peptide (PN361) matching a C-terminally amidated peptide precursor from *Araneus ventricosus* identified in a genomic study (Kono et al., 2019). This precursor has 70% sequence identify with the prohormone-1 like precursor from the honeybee *Apis mellifera* (UniProt P85798) which is believed to be cleaved to form three short peptides with neuronal activity. Another short peptide, PN366 identified in F18 and F28–F30, matches a neuropeptide in the sea slug *Aplysia californica* (UniProt P06518). Larger proteins were also detected in some fractions; for example, F18 and F31 contained a fragment at 58% and 70% total fraction components, respectively, matching a zinc metalloprotease from the nematode *Caenorhabditis elegans* (UniProt 55112) which contains a peptidase family M12A domain.

### Diversity of neuroactive peptides in *P. nigriventer* venom

The cysteine-rich scaffolds of venom peptides identified in this study were compared to the classification previously proposed for *P. nigriventer* venom peptides (Diniz et al., 2018) (Figures 3C, 4). Peptides in fractions displaying inhibitory properties corresponded to scaffolds I, II, IV, V and VIII, as well as unnamed scaffolds, while peptides in fractions with activation properties comprised mostly the scaffold V. All of these scaffolds are inhibitor cystine knot motifs, except for scaffold IV which had the highest level in F26 represented by the peptide PN350.

**FIGURE 5:**
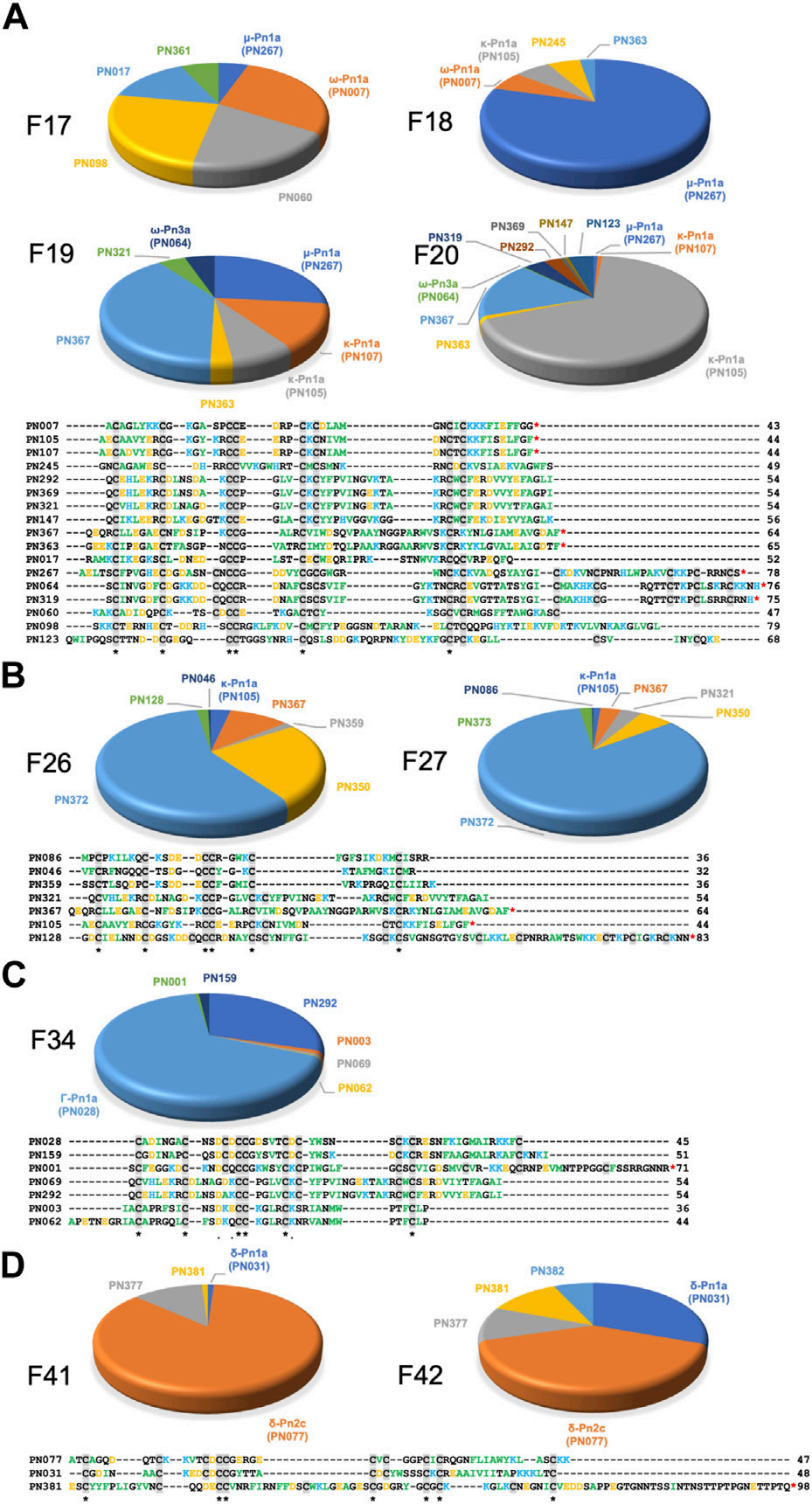
Venom peptide content of highly neuroactive RP-HPLC fractions from the venom of *P. nigriventer*. **(A)** Identification of the cysteine-rich peptides and proteins in fractions 17–20 displaying potent inhibition of neuronal Na_V_ and Ca_V_2 channels. Positively and negatively charged residues are coloured blue and orange, respectively, hydrophobic residues are green, and cysteines are highlighted in grey box. **(B)** Identification of the peptide and protein content of the fractions 16 and 27 displaying potent inhibition of neuronal Na_V_, Ca_V_1 and Ca_V_2 channels. **(C)** Identification of the peptide and protein content of the fraction 34 displaying potent inhibition of neuronal Na_V_, Ca_V_1 and Ca_V_2 channels. **(D)** Identification of the peptide and protein content of the fraction 34 displaying potent activation of neuronal Na_V_ and Ca_V_2 channels. Sequences labelled with a red asterisk (*) at the C-terminal are likely C-terminally amidated.

Neuroactive peptides with greater hydrophilicity (i.e., those with short RP-HPLC retention times) showed pharmacological properties reminiscent of known spider-derived μ-toxins (F17 and F18) and ω-toxins (i.e., inhibition of Ca_V_1 and Ca_V_2 channels by F19 and F20) (Figures 2C, 5A). Major components driving those bioactivities were the pharmacologically characterised peptides μ-CNTX-Pn1a, ω-CNTX-Pn1a and ω-CNTX-Pn3a, as well as additional peptides with unknown activity (Figure 4A). As the hydrophilicity of the peptides decrease (i.e., peptides with long RP-HPLC retention times), persistent Ca_V_2 inhibition was observed with maximum inhibitory activity in F26 and F27, and with the additional peptide ω-CNTX-Pn4a detected in F24 (Figures 2C, 3B, 5B). Interestingly, venom peptides characterized as K_V_ modulators, such as κ-CNTX-Pn1a, were detected in fractions displaying strong inhibition of calcium influx with potential μ- and ω-pharmacology (fractions 26 and 27); it was not clear if the observed bioactivity was associated to the modulation of K_V_ channels, or to other unexplored peptides in these fractions.

Neuroactive peptides presenting more hydrophobic structures showed properties of μ and ω-peptides, but with preference for Ca_V_2 channels as observed for fraction 34 in which the peptide Γ-Pn1a is the major component, consistent with its previously observed modulation of multiple cation channels (Paiva et al., 2016); and of δ-peptides as observed in fractions 41 to 45, in which major components included the peptides δ-Pn1a and δ-Pn2c (Figures 2C–F, and Figures 5C, D). Notably, the main components of some of the most neuroactive fractions are peptides with unexplored bioactivity, e.g., fraction 26 (Figures 2C, 4, 5).

### Pharmacological groups

**FIGURE 6:**
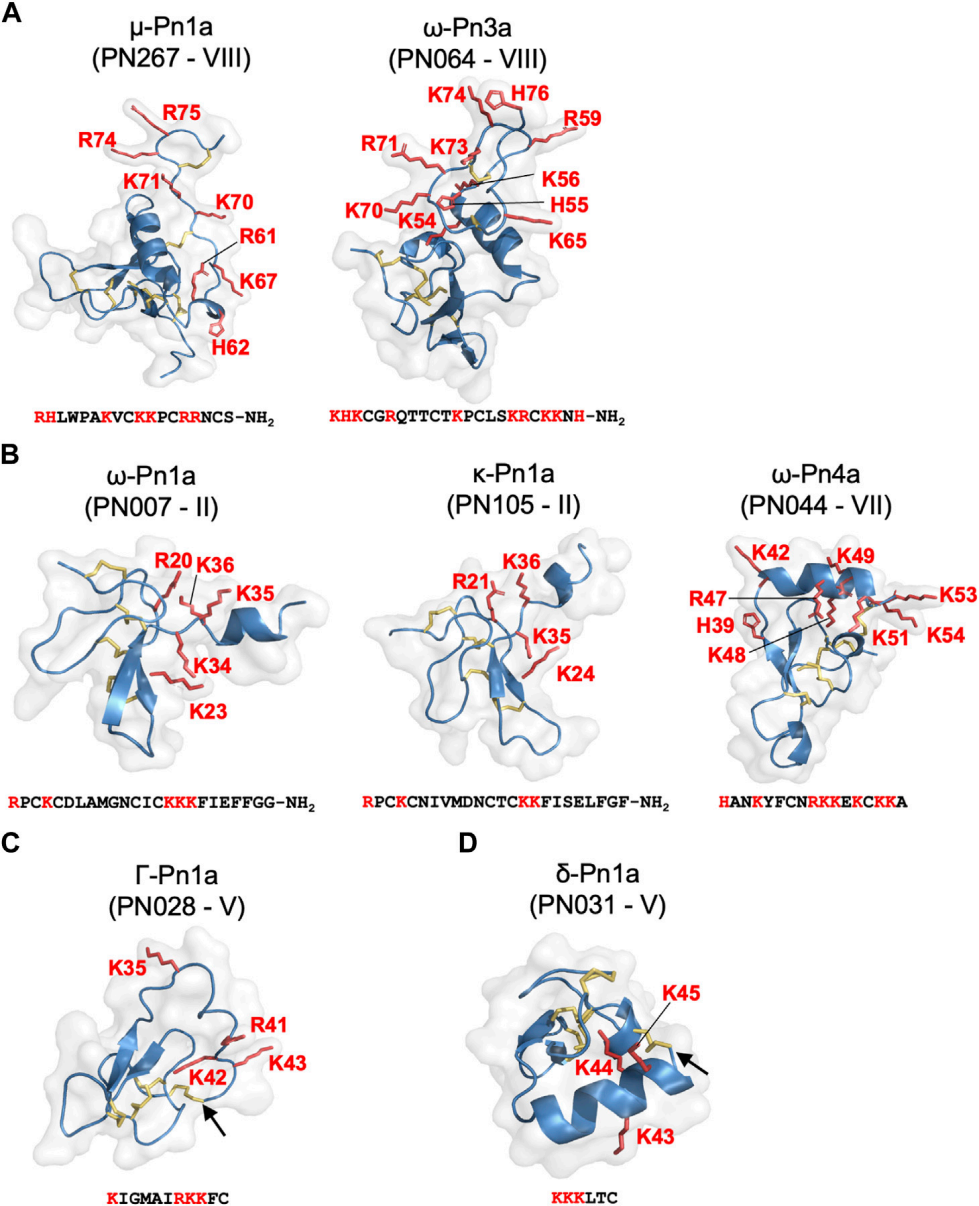
Pharmacological groups identified in the most active venom fractions highlighting the “KR electrostatic trap” pharmacophore common to spider toxins that modulate the activity of ion channels. **(A)** Group 1 is represented by μ- and ω-spider-venom peptides with large and complex type VIII scaffold. **(B)** Group 2 is represented by κ- and ω-spider-venom peptides with type II and VII scaffolds. **(C)** Group 3 is represented by γ-spider-venom peptides with type V scaffold. **(D)** Group 4 is represented by δ-spider-venom peptides displaying a type V scaffold. K and R residues located in the C-terminal region of these peptides and grouped on a positively charged face are highlighted in red in the sequences and in red tubes in the corresponding 3D structures. Arrows shows the cysteine-bridge connection forming the cyclic peptide structures predicted for PN028 and PN031.

Our approach allowed classification of *P. nigriventer* venom peptides into four major groups based on their bioactivity (Figure 6; Table 1). **Group 1** is comprised of μ and ω peptides with scaffold type VIII and more hydrophilic properties as they eluted between F17 and F21. As representatives from this group, μ-CNTX-Pn1a and ω-CNTX-Pn3a have a potential “KR electrostatic trap”, a pharmacophore described in spider-venom peptides that modulate ion channels (Hu et al., 2021; Wisedchaisri et al., 2021), in their primary and tertiary structures (Figure 6A). This pharmacophore is likely composed of residues R61, K67, K70, K71, R74 and R75 in μ-CNTX-Pn1a and residues K54, K56, R59, K65, K70, R71, K73 and K74 in ω-CNTX-Pn3a. Within this group, the ω-CNTX-Pn3a homologue PN319 differs at three positions, making it an interesting candidate for further characterisation.

**TABLE 1:** Pharmacological groups identified in this study with respective pharmacological types, cysteine-rich scaffold types, and representative venom peptides described in the literature. Unexplored peptides within each group are described in Figure 5 and/or Supplementary Table S1.

Group	Pharmacology type(s)	Cysteine-rich scaffold(s)	Venom peptide representative(s)
1	μ and ω	VIII	μ-Pn1a and ω-CNTX-Pn3a
2	κ and ω	II and VII	κ-Pn1a, ω- Pn1a and ω-Pn4a
3	Γ	V	Γ-Pn1a
4	δ	V	δ-CNTX-Pn1a

**Group 2** comprises κ and ω peptides that eluted between F17 and F28, with scaffold types II and VII (Figure 6B). As representatives from this group, peptides κ-CNTX-Pn1a, ω-CNTX-Pn1a and ω-CNTX-Pn4a also contain a “KR trap” pharmacophore comprised of residues R20, K23, K34, K35 and K36 for ω-Pn1a; R21, K24, K35 and K36 for κ-Pn1a; and K42, R47, K48, K49, K51, K53 and K54 for ω-Pn4a. In this group, PN107 differs from κ-CNTX-Pn1a by only two residues and is an interesting peptide for further exploration.

**Group 3** is comprised of more hydrophobic Γ peptides that eluted in F33–F36 and possess a type V scaffold (Figures 3C–D, 6C). It is represented by Γ-CNTX-Pn1a with a potential “KR trap” comprising residues K35, R41, K42 and K43. Although Γ peptides modulate N-methyl-D-aspartate (NMDA) glutamate receptors, Γ-CNTX-Pn1a has also been reported as a β-peptide that inhibits Na_V_ channels (Paiva et al., 2016), which agrees with the results from our high-throughput ion channels assays (Figures 2C–F, 3). Interestingly, Γ-CNTX-Pn1a predicted 3D structure formed a cyclic structure in which the N-terminal cysteine formed a disulfide bridge with C-terminal cysteine (Figures 4C, 6C). These same fractions contain other ICK peptides including PN003 and PN292 with scaffold types I and II, respectively; their pharmacological targets have not been explored but they likely contribute to the strong inhibition of Ca_V_ channels by F34 (Figures 2, 3, 4).

**Group 4** is composed of very hydrophobic δ peptides that elute in F40–F45 and possess a type V scaffold (Figure 2B, 6D). It is represented by δ-CNTX-Pn1a with potential “KR trap” comprising residues K43, K44, and K45 (Figure 6D). In this group we also identified δ-CNTX-Pn2c which differs not only in primary structure but also in the scaffold V tertiary structure by presenting a non-cyclic structure compared to the cyclic structure predicted for δ-CNTX-Pn1a connected by the N- and C-terminal cysteines (Figures 4D, 6D). Beyond these known peptides, this group comprised interesting unexplored peptides such as PN032 and PN023 showing δ peptide domains and differing from Γ-CNTX-Pn1a by 12 and 11 residues, respectively.

## Discussion

Spiders are one of the most speciose venomous taxa, with >50,000 characterised species (see World Spider Catalog, https://wsc.nmbe.ch/statistics/). Their venoms are rich in neuroactive peptides that target a wide range of neuronal ion channels and receptors using mechanisms distinct from those of neurotoxins from other venomous animals such as cone snails and scorpions. The exploration of venom peptides targeting ion channels and receptors provides novel opportunities for the development of pharmacological tools to understand disease mechanisms (Cardoso and Lewis, 2018; Cardoso, 2020) as well as provision of leads for development of therapeutics (King, 2011) and bioinsecticides (Smith et al., 2013).

Spiders are classified in two major groups, or infraorders (King, 2004): Mygalomorphae, or so-called “primitive spiders”, includes the family Theraphosidae, or tarantulas, which are the most well studied spider venoms due to the large-size and long lifespan (often >20 years) of these spiders. Araneomorphae, or “modern spiders,” comprise >90% of all extant spider species, including the family Ctenidae in which *P. nigriventer* resides. Notably, despite their much greater species diversity, araneomorph venoms are underexplored compared to mygalomorphs due to their smaller size and shorter lifespan (typically 1–2 years). Our data, and those of others (Binford et al., 2009; Zhang et al., 2010; Diniz et al., 2018; Peigneur et al., 2018), showed a great diversity of both pharmacological actions and cysteine scaffolds in araneomorph venom, which may have facilitated the highly successful araneomorph radiation. Our data also suggests Araneomorphae’s venoms may be a rich source of unique venom peptides with more diverse structures and pharmacological functions and additional biotechnological and therapeutic applications to Mygalomorphae’s venoms.

The venom from *P. nigriventer* comprises many exceptional peptides drug leads under development for treating a range of complex neuro disorders (Peigneur et al., 2018). These peptides have been evaluated in pre-clinical models and demonstrated interesting therapeutic efficacy in reverting or preventing conditions for which treatments are limited or unavailable. For example, ω-Pn2a and ω-Pn4a showed efficacy in treating painful neuropathies such as fibromyalgia and chronic post-ischemia pain, respectively (Pedron et al., 2021; Cavalli et al., 2022), ω-Pn4a also improved motor movement and neuroprotection in Huntington’s disease (Joviano-Santos et al., 2022). The engineered peptide PnPP-19 derived from the venom peptide δ-Pn2a was efficacious in treating glaucoma (da Silva et al., 2020) and erectile dysfunction (Nunes da Silva et al., 2019). In our study, these therapeutic peptides showed bioactivity at neuronal Na_V_ and Ca_V_ channels, which greatly supports our investigative platform for the discovery of venom peptides useful for the development of efficacious drugs.

Investigative pipelines in venomic studies often focus on the elucidation of venom components based on their structures but lack clear strategies to investigate venom bioactivities (von Reumont et al., 2022). Investigations using fractionated venom (Cardoso et al., 2015; Cardoso et al., 2017; Estrada-Gomez et al., 2019; Cardoso et al., 2021) provides more defined biological functions than using crude venom due to the immense pharmacological diversity of venom, which often contains venom components with opposing activity as well as components that act synergistically (Raposo et al., 2016). Considering the large number of extant spiders and consequently the exceptionally large number of venom components available for investigation, high-throughput (HT) functional bioassays are essential for developing a holistic understanding of venom pharmacology, and they provide a complement to venomic studies.

A recent study by us using HT bioassays to investigate the ion channel targets of Australian funnel-web spider venoms recaptured current taxonomy and revealed potential drug targets to treat severely envenomated patients (Cardoso et al., 2022). In this present study, we also demonstrated the feasibility of applying HT functional bioassays to investigate spider venom components that mediated the activity of voltage-gated ion channels. We were able to capture all known venom components and associated bioactivities using a HT functional assay as well as several new unexplored venom peptides that warrant further exploration. This was achievable only by combining HT bioassays with transcriptomic and proteomic approaches. Although this pipeline provides a robust holistic overview of spider venoms, bioactive components are present in varying concentrations in each fraction, which may affect bioactivity through synergistic effects, and overlook the activity of less abundant components.

The complexity of the cysteine-rich scaffolds in *P. nigriventer* venom peptides unraveled in this study suggests that further exploration utilising recombinant or synthetic peptides might be challenging but essential, and these could also benefit from modern strategies utilizing HT recombinant expression or chemical synthesis (Pipkorn et al., 2002; Turchetto et al., 2017). In tandem with automated whole-cell patch-clamp electrophysiological studies, this will build a pipeline to further investigate known and new peptides in the venom of *P. nigriventer* and allow selection of candidates with biotechnological potential. The putative “KR trap” pharmacophores identified in those venom peptides warrants further exploration of the structure-function relationships of the diverse pharmacological groups found in the venom of *P. nigriventer.*

In conclusion, we demonstrated that the introduction of HT functional bioassays in venomic studies is essential to provide a more complete understanding of venom components in terms of structure and function. It also allows venom peptides to be ranked for further investigation based on their bioactivity and structural diversity, which is not possible *via* transcriptomic and proteomic studies alone. Furthermore, this study provides a guide to assist the exploration of neuroactive venoms from other animals, in particularly for the underexplored araneomorph spiders.

## Data Availability

The datasets presented in this study can be found in online repositories. The name of the repository and accession number are ProteomeXchange PRIDE repository; PXD037904.
